# Role of β-blockers in Preventing Heart Failure and Major Adverse Cardiac Events Post Myocardial Infarction

**DOI:** 10.2174/1573403X19666230111143901

**Published:** 2023-07-05

**Authors:** Nishant Johri, Prithpal S. Matreja, Aditya Maurya, Shivani Varshney, Smritigandha

**Affiliations:** 1 Department of Pharmacy Practice, Teerthanker Mahaveer College of Pharmacy, Moradabad, Uttar Pradesh, India;; 2 Department of Pharmacology, Teerthanker Mahaveer Medical College and Research Centre, Moradabad, Uttar Pradesh, India

**Keywords:** Cardiovascular disease, HFrEF, CAD, LVEF, AMI, IHD

## Abstract

β-blockers have been widely utilized as a part of acute myocardial infarction (AMI) treatment for the past 40 years. Patients receiving β-adrenergic blockers for an extended period following myocardial infarction have a higher chance of surviving. Although many patients benefited from β-blockers, many do not, including those with myocardial infarction, left ventricle dysfunction, chronic pulmonary disease, and elderly people. In individuals with the post-acute coronary syndrome and normal left ventricular ejection fraction (LVEF), the appropriate duration of beta-blocker therapy is still unknown. There is also no time limit for those without angina and those who do not need β-blockers for arrhythmia or hypertension. Interestingly, β-blockers have been prescribed for more than four decades. The novel mechanism of action on cellular compartments has been found continually, which opens a new way for their potential application in cardiac failure and other cardiac events like post-myocardial infarction. Here, in this review, we studied β-blocker usage in these circumstances and the current recommendations for β-blocker use from clinical practice guidelines.

## 1 INTRODUCTION

Cardiovascular disease (CVD) has become a worldwide cause of death in humans [1]. As of 2012, 7.4 million individuals died from ischemic heart disease (IHD), which accounted for 15% of all deaths, according to the World Health Organization (WHO) [2-4]. Numerous cardiovascular disorders can be effectively treated with β-blockers [5, 6]. Adrenergic β-receptor blockers have been used to treat CVD for many years. In the present situation, patients with CVD live longer because of advances in primary prevention, early identification, and new treatment medicines. However, chronic cardiac disease is very frequent in humans; therefore, finding the best-customized treatment for each patient is essential. When β-blockers were first identified in the 1960s as an antianginal medication, they became widely utilized for treating cardiac failure, arrhythmias, and other heart diseases. β-blockers are considered for patients with cardiac failure who have suffered from acute myocardial infarction [7, 8]. It is now known that β-blockers have effects beyond competing with catecholamines on these receptors.

β-blockers decrease myocardial workload by lowering hypertension and heart rate to reduce oxygen demand [9, 10]. They decrease the catecholamine level, lowering myocardial ischemia, and reducing the size of the infarct, preventing definitive infarction in acute coronary syndromes [11]. When β-blockers are used early in acute myocardial infarction, sufficiency and late reinfarction rates reduce ventricular and supraventricular arrhythmias. β-blockers decrease the need for additional antiarrhythmics, chest pain, and sudden cardiac death [12, 13].

Treatment of CVD patients depends on the severity of the diseased condition. Traditional β-blockers like atenolol and metoprolol tend to have more metabolic adverse effects than newer medicines like carvedilol and nebivolol, which have higher selectivity or vasodilating characteristics.

Clinical recommendations discourage using β-blocker drugs in noncardiac surgery due to a lack of data [14-16]. Employing β-blockers in relevant clinical studies did not reduce perioperative myocardial infarctions, strokes, and death due to CVD [17]. The REACH (Reduction of Atherothrombosis for Continuous Health) registry found no link between β-blockers and side effects in persons with stable coronary arteries [17]. Fig. (**1**) shows that β-blockers improved patients' cardiovascular illness.

Contrary to previous research, no evidence has been found that β-blockers lower the risk in adults with stable ischemic cardiac disease [18-21]. However, assessment of patients is required who undergo noncardiac surgery for the benefits of β-blockers against the risk of bradycardia and hypotension [22, 23]. Regardless of their cardiovascular risk profile, patients recovering from an acute myocardial infarction (AMI) should be offered β-blockers because early clinical trials revealed that long-term usage of these medications reduced mortality [24-27].

β-blockers may be used long-term to prevent infarction, control ischemia, and improve survival in chronic stable ischemic heart disease. β-blockers may be continued in patients with decreased LVEF after AMI and chronic heart failure in functional class II-IV. They should be administered early in AMI, except in low-risk patients. β-blockers minimize the incidence of stroke and death in the acute phase of MI.

## 2 MECHANISM OF ACTION OF β-BLOCKERS ON HEART

Regulation of cardiovascular homeostasis is impossible without β-adrenergic receptors [28-30]. According to molecular pharmacology, three distinct types of β-receptors can be found in various tissues [31-34]. β1, β2, and β3 receptors are in the heart, smooth muscles, and adipocytes, respectively [35-39]. Endothelial and cardiomyocyte cells both have β3-adrenoceptors. As all β-blockers inhibit the receptor, they effectively neutralize the effects of catecholamines [40]. An analogous structure to β1-adrenoceptor that of G-protein coupled receptor (GPCR) is seen in the β2-adrenoceptor. The activation of the β-adrenoceptor causes a rise in intracellular cAMP, as shown in Fig. (**2**) [41-46]. The sarcoplasmic reticulum releases more calcium when Protein Kinase A (PKA) is present. An increase in calcium loading favorably affects the heart's pumping capacity. PKA phosphorylates troponin I and phospholamban, which increases sarcoplasmic reticulum calcium reuptake, resulting in the lusitropic (rate of myocardial relaxation) effect. The enhanced contraction of the cardiac muscle cell arises from rising cytosolic calcium levels. Antidepressants prevents functioning of the adrenoceptors [47, 48]. Table **1** [49-56] shows a list of β-blockers widely used in different cardiovascular events.

## 3 BENEFICIAL EFFECTS OF ß-BLOCKERS IN HEART FAILURE

As early as the turn of the twenty-first century, β-blockers were found to provide significant benefits for people suffering from heart failure (HF). β-blockers shows considerable effect in patients with systolic dysfunction. β-blockers are suggested for patients with chronic cardiac failure, although they are rarely used in clinical practice [57, 58].

However, no evidence of benefit was found in the group in a meta-analysis study on patients with atrial fibrillation. Differences in prognosis and heart rate between sinus rhythm and ventricular fibrillation may relate to this differentiation, but it may also be due to consequences such as myocardial fibrosis that could affect treatment efficacy; however, available data are scarce [59, 60]. More RCTs are needed to fill this data gap and the relation of renal failure in HF patients [61]. β-blockers reduce mortality and readmissions in reduced ejection fraction heart failure patients (HFrEF) [62, 63]. Additionally, β-blockers are recommended for patients with stable HFrEF (Class I, Level A). There are four licensed medications for HFrEF Bisoprol, Carvedilol, Metoprolol, and Nebivolol [64, 65].

**Fig. (3):**
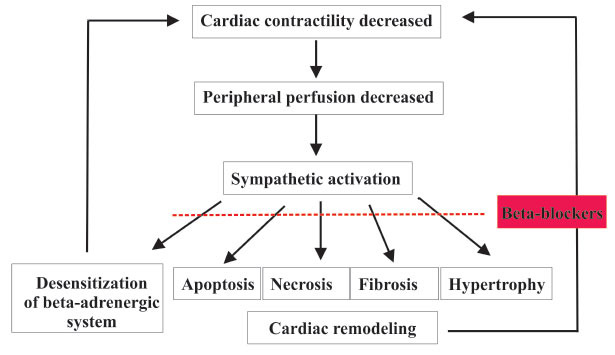
Schematic representation of cycle showing sympathetic activation in chronic heart failure.

As a result of decreasing output of the heart in chronic heart failure, numerous neurohormonal systems become activated to keep blood flowing. The adrenergic nervous system (ANS) is one of the most effective systems in the body [66, 67]. The β-adrenergic system becomes less responsive to agonist stimulation due to persistent sympathetic activation. A decrease in adrenergic activity may affect the contractility of the heart during exercise, although ventricular remodeling may be more significant in starting cardiac failure [68-70]. In both cases, reduced ventricular contractility results in decreased perfusion of the extremities. As a result, the sympathetic nervous system's compensatory activation creates a vicious cycle that eventually leads to decreased cardiac function and heart failure (Fig. **3**). To effectively treat cardiac failure, it is critical to use a blocker to break the cycle by blocking the consequences of activation of the sympathetic nervous system [71]. Long-term β-blocker use reduces the risk of death and reinfarction after MI. β-blocker eligibility should be reviewed often in MI/HF patients [72].

## 4 PATIENTS WITH ACUTE CORONARY SYNDROMES ON BETA-BLOCKER THERAPY

The 2014 AHA/ACC Guideline for the Management of Patients with Non-ST-Elevation Acute Coronary Syndromes (ACS, Class IIa, Level of Evidence C) recommends continuing β-blocker therapy in patients with a normal left ventricular function who have ACS [73]. It is recommended that all patients with ST-segment elevation MI who have no contraindications continue to take β-blockers during and after hospitalization. The AHA/ACCF 2011 update suggests three years of β-blocker therapy for this subset (Class I, Level of Evidence B). People who do not have heart failure or high blood pressure should not be given long-term beta-blocker medication [74]. For up to three years, people with heart failure can take β-blockers (Class IIa, Level of Evidence B). Pre-modern medical and reperfusion research is the basis of these recommendations. After a MI, individuals with an LVEF of less than 40% are only given long-term β-blockers according to the 2015 ESC guidelines for treating acute coronary syndromes without persistent ST-segment elevation because there have been no recent randomized trials of β-blocker medication in individuals with intact LV function following an ACS [75].

## 5 PRESCRIBING β-BLOCKERS POST-MYOCARDIAL INFARCTION

Several studies showed that β-blockers were recommended for reperfusion therapy before the widespread use of coadjutant antithrombotic and cholesterol-lowering pharmaceutical therapy (anticoagulation and antiplatelet medication). Old prospective randomized studies have shown that β-blockers improve outcomes post-myocardial infarction and lower mortality in about 20% of cases. Many patients in these trials had MI with joint left ventricular dysfunction in the 1980s; however, these trials were done before the introduction of advanced techniques like reperfusion therapy. These patients had large MIs.

Patients with MI now have a far better prognosis thanks to improvements in invasive care and pharmaceutical therapy. If β-blockers are still advantageous in the new scenario without heart failure or left ventricular dysfunction, the question is whether the benefits of these drugs are well established in patients with a lower LVEF (40%). Patients with MI with intact LVEF treated according to current guidelines, such as complete revascularization, reperfusion, and effective antithrombotics have no evidence that maintenance β-blockers are helpful. No mortality benefit was found in the reperfusion period when studies were stratified into reperfusion and postreperfusion periods [76].

When adjusting for other risk variables, individuals with reduced LVEF who adhered to β-blocker medication were more likely to survive and have a lower chance of developing heart failure four years after the index incident than those with maintained LVEF. In recent studies of β-blockers in MI, only acute administration of β-blockers during ST-segment–elevation MI (STEMI) has been studied. Intravenous administration of metoprolol during an ongoing anterior STEMI reduced infarction size [77], decreased microvascular obstruction and reperfusion injury, and improved long-term LVEF in the METOCARD-CNIC trial (Metoprolol in Cardioprotection During an Acute Myocardial Infarction) was found [78].

Due to the delay between diagnosis and reperfusion, metoprolol positively affects patients with ischemic heart disease. Recent research shows that the acute intravenous administration of metoprolol after an ongoing STEMI does not have the same positive effects on the cardiovascular system as other β-blockers. There was no way to evaluate the value of long-term β-blockers in the METOCARD-CNIC trial because they were given to all patients on day one. Since reperfusion, more studies have focused on short-term β-blocker treatment in STEMIs [79].

Before the widespread use of reperfusion therapy and coadjutant antithrombotic and cholesterol-lowering pharmacological therapy, β-blockers were suggested in several trials after a MI (anticoagulation and antiplatelet medication). Long-term treatment with β-blockers post-myocardial infarction improves outcomes and reduces mortality by around 20%, according to old prospective randomized studies. Before contemporary reperfusion and medical care, many patients in these trials experienced central MI with common left ventricular failure.

Invasive care and pharmacological therapy have improved the outcome for MI patients. This raises the question of whether β-blockers are still beneficial in the absence of heart failure or LV dysfunction (40%). β-blockers are not beneficial in patients with MI who have an intact LVEF and receive current treatment recommendations, such as complete revascularization, reperfusion, effective antithrombotics, and intensive cholesterol lowering. No mortality advantage was reported when studies were split into pre- and post-reperfusion.

After adjusting for other risk variables, patients with impaired LVEF who took β-blockers had a significantly longer survival and a lower probability of developing heart failure four years after the index incident. Over the last few years, only acute administration of β-blockers in MI studies has been investigated. Intravenous metoprolol during continuing anterior STEMI lowers infarction size, microvascular blockage, and reperfusion injury [80] and improves long-term LVEF. Metoprolol is not as effective as other beta-blockers when given intravenously after a STEMI. The METOCARD-CNIC trial could not assess the value of long-term β-blockers because they were administered to all patients on day one of treatment. Studies on short-term β-blocker treatment in STEMIs have increased since reperfusion (Table **2**).

## 6 CARDIAC FAILURE WITH A DECREASED EJECTION FRACTION

The outcomes of major randomized, placebo-controlled studies are the critical source of guidance for investigating nebivolol (SENIORS), bisoprolol (CIBIS-II), metoprolol (MERIT-HF), and carvedilol (COPERNICUS). In these trials, drugs lowered the risk of death and hospitalization (Table **3**) [81]. A meta-analysis of randomized studies found that using β-blockers decreased mortality risk by over 30%. β-blockers also lowered mortalities from cardiovascular disease and sudden death. There were no significant variations found in the study when comparing with individual β-blockers [82].

## 7 CONTRAINDICATIONS AND SIDE EFFECTS

β-blockers cause bradycardia, hypotension, cold extremities, bronchospasm, headaches, increased insulin resistance, and insomnia. Cardio selective β-blockers should be used while treating COPD; starting at a low dose and increasing gradually. Asthmatic patients are not given β-blockers. These medications should only be taken by a doctor or other qualified healthcare professionals [83]. Studies have not shown that β-blockers cause depression, weight gain, and headaches compared to the control group. Some adverse effects have been reported, such as a slight increase in the chance of hyperglycemia, diarrhea, and dizziness and the risk of claudication and bradycardia [84].

## CONCLUSION

In individuals with sinus rhythm, heart failure, and a poor ejection fraction, beta-blockers reduce the risk of death as well as the need for hospitalisation. On the other hand, it would appear that beta-blockers have little to no effect in a variety of medical conditions. Even though they are often used in everyday clinical practise, the overall clinical result of beta-blockers appears to be highly dependent on the clinical context in which they are administered. A randomised controlled trial is required in order to obtain better understanding of how blockers can now be utilised to treat heart failure and MI. Further, research is necessary on a global scale to assess the suitability of blockers, as well as their limitations and possible alternatives.

## Figures and Tables

**Fig. (1):**
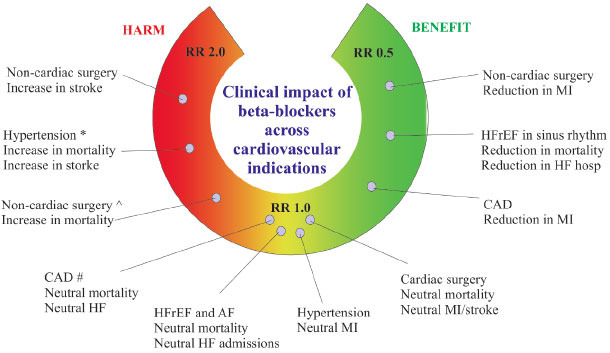
Overview of β-blockers in CVS. *In comparison to other drugs. ^ In clinical trials without risk of bias. # Drug undergoing ref perfusion in contemporary trials. **Abbreviations:** HF, heart failure; CAD, coronary artery disease; AF, atrial fibrillation; HFrEF, heart failure with reduced ejection fraction; MI, myocardial infarction; RR, risk ratio.

**Fig. (2):**
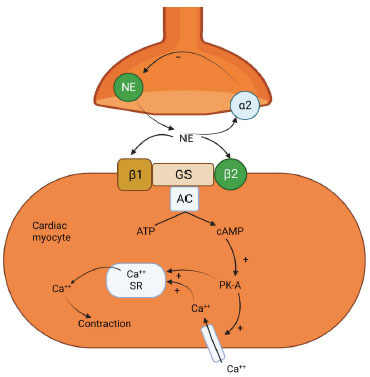
Schematic representation of the mechanism of action of beta-blockers.

**Table 1:** Characteristics profile and comparison between clinical significance of beta blockers in heart.

**Drug**	**Receptor Specificity**	**Intrinsic Sympath-Omimetic Activity**	**Usage in CVD**	**References**
Acebutolol	β1	Yes	Chronic stable angina; tachyarrhythmia	[50]
Atenolol	β1	No	Chronic stable angina; tachyarrhythmia; myocardial infraction	[51]
Carvedilol	β1, β2, ⍺1	No	Heart failure	[52]
Labetalol	β1, β2, ⍺1	No	Heart failure; Chronic stable angina; tachyarrhythmia; myocardial infraction	[53]
Metoprolol	β1	No	Heart failure; Chronic stable angina; tachyarrhythmia; myocardial infraction	[54]
Pindolol	β1, β2	Yes	Indicated in coronary artery disease and heart failure, inappropriate sinus tachycardia	[55]
Esmolol	β1	No	Control rate of atrial flutter/atrial fibrillation	[56]
Propranolol	β1	No	Chronic stable angina; tachyarrhythmia; thyrotoxicosis; myocardial infraction	[57]

**Table 2:** Current beta-blocker recommendations after MI.

**Recommendation**	**Class of Recommendation**	**Level of Evidence**
**2014 AHA/ACC Guidelines for Non-ST-Elevation Acute Coronary Syndromes**		
In the absence of HF, low output status, risk for cardiogenic shock, or other contraindications to beta-blockade, begin oral beta-blockers within the first 24 hours.	I	A
For patients with simultaneous ACS without ST-segment elevation, stabilised HF, and impaired systolic function, sustained-release metoprolol succinate, carvedilol, or bisoprolol is suggested.	I	C
Patients with ACS who do not have ST-segment elevation but have normal LV function can safely continue beta-blocker medication.	IIa	C
**The 2013 ACCF/AHA ST-Elevation Myocardial Infarction Management Guideline**		
With ST-segment elevation MI, begin oral beta blockers within the first 24 hours of onset for patients without HF, low-output states or other contraindications to beta-blockade.	I	B
All patients with ST-segment elevation MI who have no contraindications to beta-blocker treatment should continue taking them during and after hospitalisation.	I	B
**Treatment of Patients with Coronary and Other Atherosclerotic Vascular Disease as Secondary Prevention and Risk Reduction by the AHA/ACCF: 2011 Update**		
All patients who have had a MI or ACS and have normal LV function should begin and continue beta-blocker therapy for three years.	I	B
Chronic beta-blocker medication is suitable for all patients with healthy left ventricles who have undergone a MI or ACS over the course of at least three years.	IIa	B

**Table 3:** Output of randomized control trials (RCT) in patties with cardiac failure and decreased ejection fraction [82].

**Trial**	**β-Blocker Drugs**	**N**	**Mean Duration**	**EF**	**Primary Outcomes**
SENIORS	Nebivolol	2128	21 months	35%	Hospitalization for cardiovascular disease or death
COPERNICUS	Carvedilol	2289	10.4 months	25	The combined risk of hospitalization for cardiovascular disease or death
CIBIS-II	Bisoprolol	2647	1.3 years	35%	Mortality
MERIT-HF	Metoprolol	3991	1 year	40%	Combined all-cause hospitalization or mortality

**Abbreviations:** SENIORS - Study of Effects of Nebivolol Intervention on Outcomes and Rehospitalization in Seniors With Heart Failure; COPERNICUS - Carvedilol Prospective Randomized Cumulative Survival; CIBIS-II - Cardiac Insufficiency Bisoprolol Study, MERIT-HF - Metoprolol CR/XL Randomised Intervention Trial in Congestive Heart Failure; EF - Ejection fraction, CV - Cardiovascular, HF - Heart failure.

## LIST OF ABBREVIATIONS

AMI: Acute Myocardial Infarction
LVEF: Left Ventricular Ejection Fraction
CVD: Cardiovascular Disease
IHD: Ischemic Heart Disease
